# The central mechanisms of electroacupuncture at LR3 in the treatment of spontaneous hypertension: a PET and mRNA transcriptome study

**DOI:** 10.3389/fcvm.2024.1358426

**Published:** 2024-08-21

**Authors:** Jing Li, Chong Peng, Kejie He, Yumei Wang, Xinsheng Lai

**Affiliations:** ^1^ Integrative Cancer Centre, The First Affiliated Hospital of Guangzhou University of Chinese Medicine, Guangzhou, Guangdong, China; ^2^ Postdoctoral Research Station, Guangzhou University of Chinese Medicine, Guangzhou, Guangdong, China; ^3^ Clinical School of Acupuncture and Rehabilitation, Guangzhou University of Chinese Medicine, Guangzhou, Guangdong, China; ^4^ Department of Hepatobiliary Disease, The First Affiliated Hospital of Guangzhou University of Chinese Medicine, Guangzhou, Guangdong, China; ^5^ Lingnan Medical Research Center, Guangzhou University of Chinese Medicine, Guangzhou, Guangdong, China; ^6^ Department of Acupuncture and Rehabilitation, The First Affiliated Hospital of Jinan University, Guangzhou, Guangdong, China; ^7^ Department of Rehabilitation, Shenzhen Bao'an Traditional Chinese Medicine Hospital Group, Shenzhen, Guangdong, China

**Keywords:** spontaneously hypertensive rat, hypertension, electroacupuncture, PET-CT, mRNA transcriptome

## Abstract

**Objective:**

To reveal the efficacy and potential mechanisms of electroacupuncture (EA) in treating hypertension.

**Methods:**

Male spontaneously hypertensive rats (SHRs) were randomly assigned to the SHR group, EA group, and Sham-EA group, with Wistar-Kyoto rats (WKY) as the normal control group. SHRs in the EA group received electroacupuncture at the bilateral Taichong (LR3) acupoints for 7 consecutive days. Evaluation of systolic blood pressure (SBP), diastolic blood pressure (DBP), mean arterial pressure (MAP), and heart rate (HR) was conducted. Positron emission tomography-computed tomography (PET-CT) was employed to explore the active brain regions associated with acupuncture-induced blood pressure reduction. Furthermore, mRNA expression profiling was analyzed in the active brain regions to identify differentially expressed genes, and quantitative polymerase chain reaction (qPCR) was used to validate the mRNA expression of differentially expressed genes in the active brain region.

**Results:**

EA reduced elevated SBP, DBP, MAP and HR in SHR. PET-CT revealed that EA decreased glucose metabolism in the hypothalamus. Genomic analysis suggested that, compared to the SHR group, the differentially expressed genes in the hypothalamus of the EA group included Nr4a1, Sirt1, Trh, GPR88, Cck, and Th. EA downregulated the mRNA expression of Th, Trh, Gpr88, and Nr4a1, while upregulating the expression of Sirt1 and Cck at the mRNA level.

**Conclusion:**

EA may exert a unique antihypertensive effect in the hypothalamus of SHR, involving the modulation of sympathetic nerve activity, neuroinflammation, and oxidative stress response.

## 1 Introduction

High blood pressure(BP) is defined as a sustained systolic blood pressure(SBP) of at least 130 mm Hg or diastolic blood pressure(DBP) of at least 80 mm Hg, affecting approximately 116 million adults in the United States and over 1 billion adults globally (1). If left uncontrolled, hypertension increases the risk of cardiovascular diseases, stroke, kidney diseases, and negatively impacts the health and quality of life of patients (2, 3). Over the past decade, hypertension-related mortality has increased by 34.2%; in 2020, hypertension was a major cause or contributing factor to over 670,000 deaths in the United States, accounting for 20% of the total deaths (4). Despite being a modifiable risk factor, only 24% of hypertensive adults have their BP adequately controlled (5), indicating a significant unmet need for effective hypertension management (4).

Acupuncture, a non-pharmacological traditional Chinese medicine treatment, has been used for various diseases and conditions (6–10), including hypertension (11, 12). Studies suggest that the antihypertensive efficacy of acupuncture has been repeatedly validated at both clinical and basic research levels (13–15). Acupuncture is reported to affect sympathetic nerve excitability by stimulating specific acupoints, leading to a steady reduction in BP (16). Importantly, acupuncture has fewer adverse reactions compared to Western medicine, making it an effective alternative for BP reduction. However, the potential central mechanisms underlying acupuncture's BP improvement have not been fully revealed. The use of nuclear medicine-related technologies to reveal the mechanisms of acupuncture has significant research importance (17–20).

Recently, researchers have discovered that the efficacy of acupuncture exhibits acupoint-specific characteristics, acting through the activation of corresponding target brain regions (21). Positron emission tomography (PET) is a non-invasive imaging technique commonly used to measure the cerebral glucose metabolic rate and has been applied to provide unique insights into neuropathological changes in specific brain regions before and after acupuncture treatment for diseases such as hypertension (22), Alzheimer's disease (23) and stroke (24). Some researchers have employed PET-CT technology to understand the working mechanism of acupuncture, indicating that different brain regions were stimulated by acupuncture at different acupoints (25–27). However, the target brain regions and potential molecular mechanisms of acupuncture at Taichong (LR3) for reducing BP have not been revealed. Therefore, this study initially used PET-CT technology to identify the target brain regions for acupuncture at LR3 in reducing BP. Subsequently, RNA sequencing technology was employed to identify differentially expressed genes that acupuncture regulates in this brain region. Further functional annotation and bioinformatics analysis of these differentially expressed genes were conducted to determine the biological processes, pathways, and molecular functions they were involved in. Finally, the expression levels of these differentially expressed genes were validated using quantitative polymerase chain reaction (qPCR). This promising PET-CT and transcriptome study on acupuncture's BP reduction will contribute to our understanding of the central molecular mechanisms of acupuncture in hypertension treatment.

## 2 Materials and methods

### 2.1 Animals

Thirty male spontaneous hypertensive rats (SHRs) and 10 Wistar Kyoto rats (WKY) (10 weeks old, SPF grade, weight 200–220 g) were obtained from the Vital River Laboratory Animal Technology Co. Ltd, Beijing, China. Animals were housed in a temperature (24 ± 2°C) and humidity (55 ± 5%) controlled environment with a 12-h light/dark cycle and had free access to food and water. All experimental protocols strictly complied with international ethical guidelines and the Guidelines for the Care and Use of Laboratory Animals of the Ministry of Science and Technology of the People's Republic of China, and were approved by the Experimental Animal Ethics Committee of Guangzhou University of Traditional Chinese Medicine.

### 2.2 Grouping and electroacupuncture (EA) treatment

**Figure 1:**
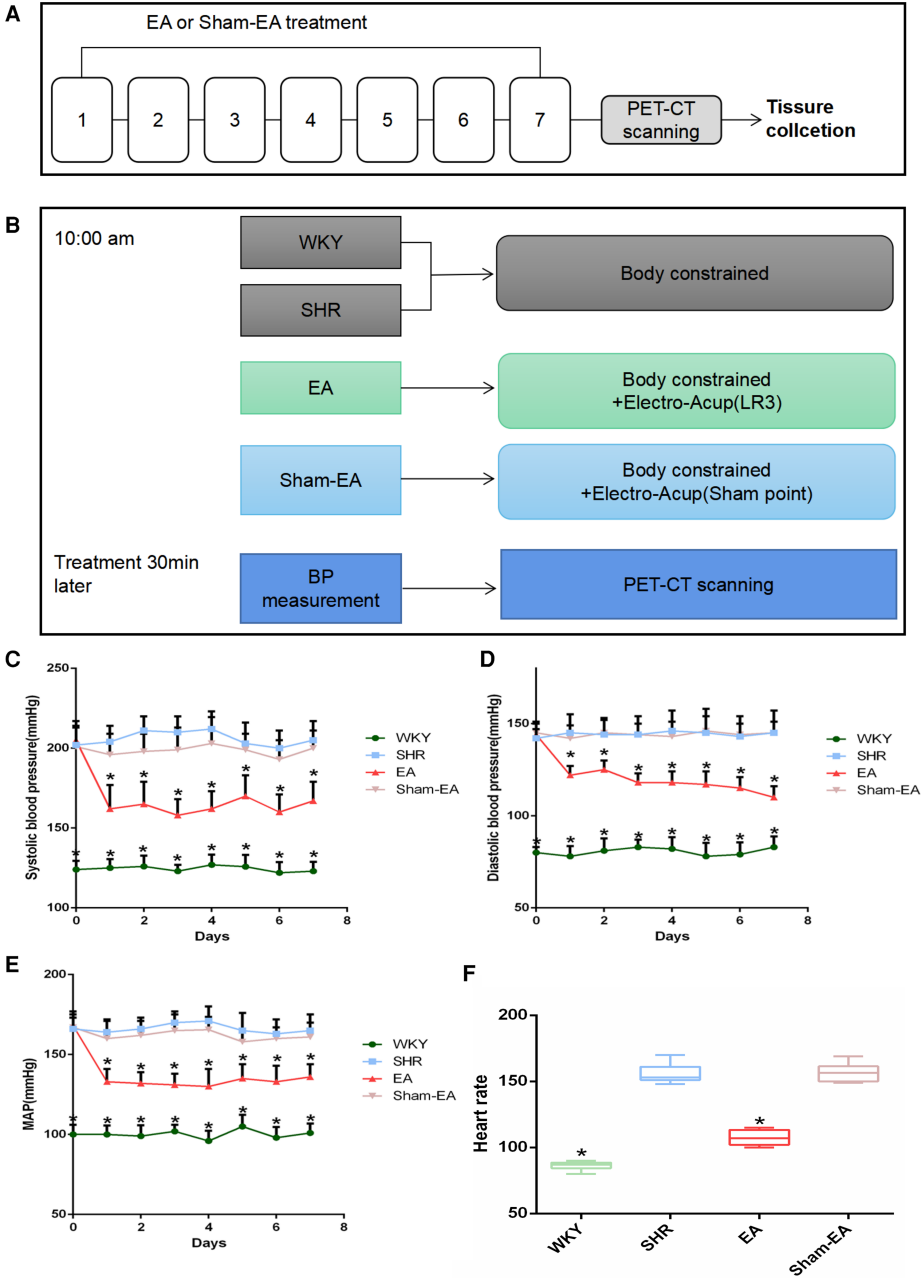
Experimental flow chart and blood pressure(BP) lowering effect induced by electroacupuncture(EA) in SHRs. **(A)** Schematic illustrating the experimental design of the EA lowering BP paradigm. From the 1st day to the 7th day, the EA group and the Sham-EA group were treated with EA every day, and the BP of the rats in the 4 groups was recorded 30 min after the treatment. After BP was measured on day 7, rats were subjected to PET-CT scans, then tissues were collected immediately. **(B)** Treatments were performed as described in “Materials Methods”. **(C)** Regulatory effect of EA on systolic blood pressure(SBP), *n* = 10. **(D)** Regulatory effect of EA on diastolic blood pressure(DBP), *n* = 10. **(E)** Modulatory effect of EA on mean arterial pressure(MAP), *n* = 10. **(F)** Monitoring the regulation of EA on heart rate(HR) in 30 min after 7 consecutive days of treatment, *n* = 10. SBP, DBP, and MAP were presented as mean ± SD and analyzed using repeated-measures ANOVA. HR data were presented as median ± interquartile range and analyzed using the Kruskal-Wallis test, *P < 0.05.

The animal experiment process design is shown in Figure 1, and all experiments were performed between 10:00–16:00. In order to observe the effect of EA on the BP of SHRs, SHRs were randomly divided into 3 groups: SHR group (*n* = 10), EA group (*n* = 10), Sham-EA group (*n* = 10), and WKY was used as the control group (WKY group, *n* = 10). Both EA and sham-EA groups received EA intervention, while WKY group and SHR group did not receive any EA treatment and only performed the same grasping and restraint operations.

Among them, the SHR group and WKY group did not receive any EA treatment, but only performed the same grasping and restraint operations. The EA group used LR3, while the Sham-EA group used the depressed sham-acupoint located at the junction of the third and fourth metatarsals on the dorsum of the foot. A disposable stainless steel acupuncture needle (length 13 mm, diameter 0.25 mm, SUIXIN, Suzhou, China) was inserted into LR3 or sham-acupoint 3–4 mm, and then the Han's acupoint neurostimulator (HANS-200, Nanjing, China) was used at a frequency of 2 Hz and an intensity of 1 mA for EA. Each treatment lasts 10 min for 7 days.

### 2.3 BP measurement

As previously described, rats were anesthetized using 3% isoflurane in 70/30% medical air/oxygen via a rodent ventilator (28). Before the measurement, the laboratory temperature was maintained at 24–26°C, ensuring a quiet operating environment and avoiding strong light exposure. Gentle handling of the rats was essential to prevent excessive agitation. Opened the CODA7m non-invasive BP measurement system, checked if the pressure sensor was working properly, set and calibrated the baseline of the BP measurement system, with an inflation pressure of 220 mmHg, a heating plate temperature of 36℃, and preheat for 5 min. Gently grasped the rats and placed them in the restraint device, then position rats on the heating pad so that their abdomen was in contact with the pad. Secured the sensor sleeve around the base of the tail, cover the cage with black fabric to retain heat, and preheated for 2 min. Measured the temperature of the rats' tails with a thermometer, and when the temperature reaches 34–36℃, begin BP measurement. Monitored the rats' BP using the system: DBP, SBP, mean arterial pressure (MBP), heart rate (HR), with a total of 15 cycles of measurements, and the average value was recorded as the final BP value.

### 2.4 ^18^FDG-PET scanning

All ^18^F-fluorodeoxyglucose positron emission tomography (^18^FDG-PET) images were acquired at the Animal Molecular Imaging Research Platform of Sun Yat-sen Medical College. After a 7-day treatment period, animals fasted for 24 h before obtaining PET images. 15 min before scanning, all animals received a tail vein injection of 1.5 mci/kg 18F-FDG. Five minutes before scanning, rats were anesthetized with 5% isoflurane and 100% oxygen, and the scans were performed on the Siemens Inveon PET system (Siemens, Germany). After the FDG-PET image acquisition was completed, the image was reconstructed using a 128 × 128 × 159 matrix and filtered back-projection algorithm. Images were spatially normalized and smoothed using the SPM8 toolbox called SPMratIHEP. All FDG-PET functional images were standardized to the SPMratIHEP rat brain PET template to eliminate individual differences, and then resliced into 1.0 × 1.0 × 2.0 mm^3^ voxels. In this step, automatic extraction of intracranial tissues was performed and normalized to the Paxinos and Watson rat brain atlas. Additionally, spatially normalized functional images were smoothed with a Gaussian kernel of 2 × 2 × 4 mm^3^ FWHM. Subsequently, voxel-wise statistical analysis of preprocessed images was conducted in the SPMratIHEP framework based on the general linear model. A two-sample *t*-test was performed to determine the differences in FDG signals between the two groups. Proportional scaling was applied to address global confounding factors. Regions of significant FDG changes in the rat brain were generated based on a voxel-level threshold of *p* < 0.001 and a cluster extent threshold of 100 voxels.

### 2.5 Tissue processing

After treatment on the 7th day, BP measurement and PET-CT scanning were completed, and the rats were euthanized and transcardially perfused. According to previous studies, the level of BP remained lower within 48 h after treatment (29). Hypothalamus was rapidly isolated, and hypothalamus total RNA was extracted using the RNeasy Mini Kit (75246, Qiagen, Beijing, China) according to the manufacturer's protocol. Finally, Agilent 2,100 Bioanalyzer (Agilent RNA 6000 Nano Kit, Santa Clara, California, USA) was used for quality control of total RNA.

### 2.6 RNA-seq analysis

RNA transcriptome sequencing was performed by BGI Genomics Co., Ltd., Shenzhen, China (http://www.genomics.cn/). Briefly, DNA libraries were constructed using the TruSeq Stranded mRNA Library Prep Kit (Illumina, San Diego, CA, USA) according to the manufacturer's instructions. Read 100 bp paired-end reads, sequence the DNA library on the BGISEQ-500 platform, and perform sequence data analysis.

### 2.7 Differential expression analysis

Gene expression levels were calculated using RSEM21. Differentially expressed genes(DEGs) were detected with DESeq2 as requested. DEGs were selected based on parameters with fold change ≥2 and adjusted *p*-value (*p* ≤ 0.05). Gene expression was compared between SHR and WKY groups, between EA and SHR groups, and between Sham-EA and SHR groups. Hierarchical clustering between all samples was performed using hclust. MA-plot, volcano-plot were generated using R version 3.6.0 (https://cran.r-project.org/bin/windows/base/old/3.6.0/). The overlap of up- and down-regulated gene expression between different groups was analyzed using Venn online software VENNY version 2.1 (https://bioinfogp.cnb.csic.es/tools/venny/index.html). GO and KEGG classification and functional enrichment were performed on all identified DEGs. GO and KEGG analyzes were performed using enrichment analysis and the phyper function in R version 3.6.0 (https://cran.r-project.org/bin/windows/base/old/3.6.0/).

### 2.8 Real-time PCR

Total mRNA was isolated and quantified from hypothalamus using the Magnosphere™ UltraPure mRNA Purification Kit (Cat. No. 8543, TaKaRa, Japan). The mRNA was then reverse transcribed into cDNA using the PrimeScript™ High-Fidelity RT-PCR Kit (Cat. No. R038B, TaKaRa, Japan). Quantitative RT-PCR was performed using TB Green Premix Ex Taq II (catalog numbers: RR873l, TaKaRa, Japan) on a CFX Connect Real-Time System (Bio-Rad, California, USA). β-actin was used as an internal reference gene, and relative gene expression was confirmed using the 2^−*ΔΔ*Ct^ method. PCR primers are shown in Table 1.

**Table 1:** Primer sequence information.

Gene	Primer sequences
Nr4a1	F:CCCTGAAGTTGTTCCCCTCAC
Nr4a1	R:GCCCTCAAGGTGTGGAGAAG
Sirt1	F:TAGCCTTGTCAGATAAGGAAGGA
Sirt1	R:ACAGCTTCACAGTCAACTTTGT
Trh	F:GCTCTGGCTTTGATCTTCGTG
Trh	R:CCGGACCTGGACTTTCTCC
GPR88	F:GAAGAGTGAAACCACAGGTGTGTACAC
GPR88	R:GTTTGTTTCCTCACTGGCTGAGAGTC
CCK	F:CAGCAAGCCAGGAAAGGT
CCK	R:TACTCGTATTCTTCAGCAC
Th	F:AAGCCAAAATCCACCACTTAGA
Th	R:GCTTGTATTGGAAGGCAATCTC
β-actin	F:CCATGTTCCAAAACCATTCC
β-actin	R:GGGCAACCTTCCCAATAAAT

### 2.9 Statistical analysis

All data were expressed as mean ± SD or median ± interquartile range, and analyzed using SPSS 17.0 (SPSS Inc., Chicago, USA). Normal distribution data were analyzed using one-way analysis of variance (ANOVA) or repeated measures ANOVA, followed by LSD *post hoc* test to determine intergroup differences. Kruskal-Wallis rank sum test was used to analyze non-normally distributed data, followed by Dunn's *post hoc* test to determine intergroup differences. *p* < 0.05 was considered statistically significant.

## 3 Results

### 3.1 Effect of EA on SBP, DBP, MAP and HR

To evaluate the effect of EA on hypertension, animals were treated for 7 consecutive days, and SBP, DBP and MAP were measured 30 min after each treatment. As shown in Figures 1C–E, compared with the SHR group, SBP, DBP and MAP were significantly decreased on the first day after acupuncture in LR3. Subsequently, for the next 6 days, the BP of the rats was repeatedly measured 30 min after EA treatment every day, and the BP of all SHRs decreased. Furthermore, the elevated HR was suppressed after the last treatment (Figure 1F). As expected, Sham-EA treatment had no significant effect on BP and HR.

### 3.2 PET-CT scanning in the brain of SHRs

**Figure 2:**
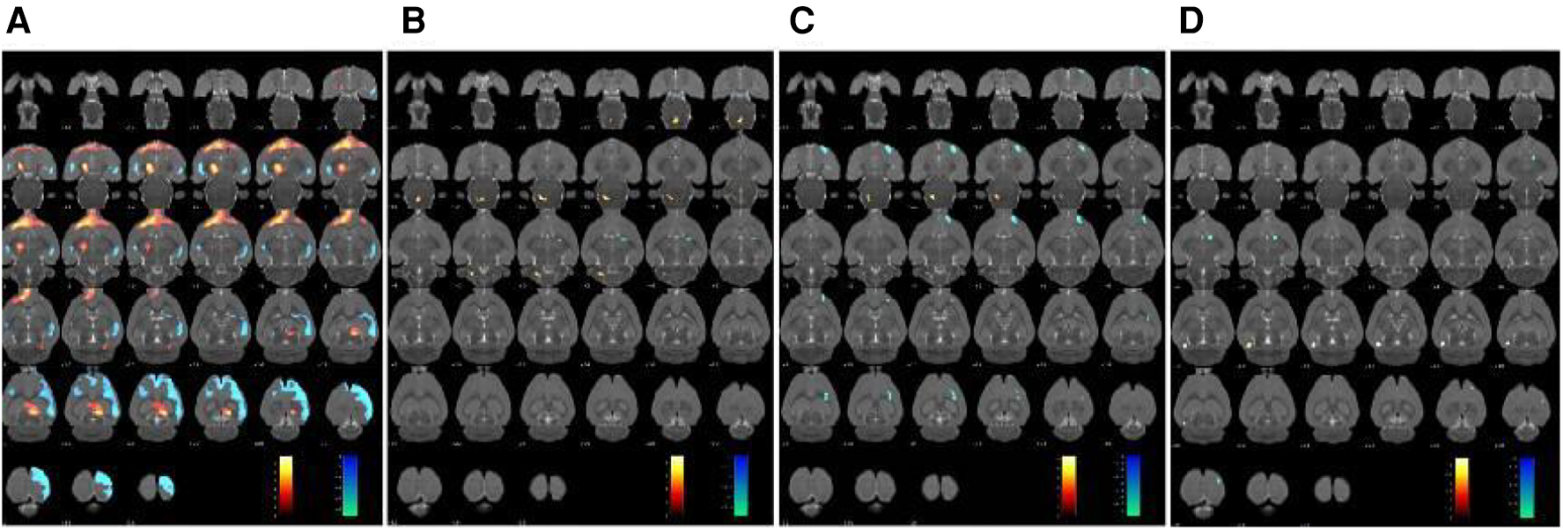
Glucose metabolism in the brain region was scanned after 7 days of treatment. The results were superimposed onto axial perspectives of the rat brain and aligned with the Paxinos and Watson rat brain atlas. **(A)** SHR group compared with the WKY group, *n* = 10. **(B)** EA group compared with the SHR group, *n* = 10. **(C)** Sham-EA group compared with the SHR group, *n* = 10. **(D)** EA group compared with Sham-EA group, *n* = 10. The color scale corresponds to the *t*-values observed for each significant voxel, where warm colors represent up-regulation of glucose metabolism, and cool colors represent down-regulation of glucose metabolism.

The changes in cerebral glucose metabolism in the SHRs brain were investigated. Compared to the WKY group, the SHR group exhibited a significant increase in glucose metabolism in the dorsal thalamus, dorsal thalamus lateral nucleus group, hypothalamus, lateral prefrontal cortex, and prefrontal cortex. However, other cerebral regions, including the sensory cortex, caudate putamen, olfactory Bulb, and visual cortex, showed a decrease in glucose metabolism (Table 2 and Figure 2A). In contrast, compared to the SHR group, the EA group showed a significant reduction in glucose metabolism in the dorsal thalamus, dorsal thalamus lateral nucleus group, and hypothalamus, while the cerebellum anterior lobe, medulla oblongata, and cerebellum posterior lobe exhibited a significant increase in glucose metabolism (Table 3 and Figure 2B). Furthermore, compared to the SHR group, the Sham-EA group displayed a significant decrease in glucose metabolism in the thalamus, medulla oblongata, and pontine tegmentum of pons, while the sensory cortex and caudate putamen showed an increase in glucose metabolism (Table 4 and Figure 2C). Finally, the EA group was compared with the Sham-EA group. The results showed that, compared to the Sham-EA group, EA downregulated the glucose metabolism in the hypothalamus, basal ganglia, and striatum, while the glucose metabolism in the corpus callosum, cerebellar hemisphere, hippocampus, and some cortical tissues was upregulated by EA (Table 5 and Figure 2D).

**Table 2:** Changes in cerebral glucose metabolism between the WKY group and the SHR group.

Anatomical	Max-T	Peak coordinates(mm)		
		*X*	*Y*	*Z*
Increased cerebral glucose metabolism				
Ventral orbital cortex	6.4924	−1.6762	3.9648	4.4421
Ventral prefrontal cortex	6.4924	−1.6762	3.9648	4.4421
Lateral orbital cortex	6.2512	−1.9434	3.8033	4.4421
Dorsal thalamus lateral nucleus group	7.8298	−1.9715	6.8136	−3.2379
Dorsal thalamus	7.8298	−1.9715	6.8136	−3.2379
Thalamus	7.6616	−1.9748	6.7923	−2.9979
Lateral prefrontal cortex	6.2512	−1.9434	3.8033	4.4421
Hypothalamus	5.0543	2.115	7.3261	−2.5179
Orbital cortex	6.4924	−1.6762	3.9648	4.4421
Prefrontal cortex	6.4924	−1.6762	3.9648	4.4421
Decreased cerebral glucose metabolism				
Olfactory Bulb	5.2385	5.2716	1.9982	−5.1579
Cingulate gyrus	5.2715	−0.31003	0.87518	2.2821
Cingulate cortex	5.2828	−0.58062	0.6924	2.5221
Parietal association cortex	5.0401	2.1747	−0.08085	−3.4779
Medial prefrontal cortex	5.2828	−0.58062	0.6924	2.5221
Caudate putamen	4.87	−5.7102	4.4282	−3.4779
Visual cortex	5.6775	5.2782	2.3329	−5.6379
Sensory cortex	6.06	3.3313	0.85868	−0.1179
Corpus callosum	6.0794	5.2749	2.8957	−5.3979
Motor cortex	6.595	1.7606	−0.0432	−2.5179

The significance was reflected by the Max_T. “*X,Y,Z*” represented the peak coordinates of the brain region's maximum effect point in Paxinos and Watson space, where the *x*-axis was midline with positive values on the right, the *y*-axis was positive ventrally and negative dorsally, and the *z*-axis was positive relative to the olfactory bulbs and negative towards the cerebellum.

**Table 3:** Changes in cerebral glucose metabolism between the EA group and the SHR group.

Anatomical	Max-T	Peak coordinates(mm)		
		*X*	*Y*	*Z*
Increased cerebral glucose metabolism				
Cerebellum anterior lobe	3.1472	−3.5999	5.2257	−11.1579
Cerebellum posterior lobe	3.3234	−1.4419	6.4993	−12.5979
Medulla oblongata	5.5108	−1.7091	7.798	−12.5979
Pontine tegmentum of pons	4.9295	−1.031	8.4847	−13.3179
Decreased cerebral glucose metabolism				
Basal ganglia	3.6155	3.2109	4.4408	−1.0779
Bed nucleus	3.1603	2.546	4.2774	−1.3179
Hypothalamus tuberal region	3.6155	3.2109	4.4408	−1.0779
Hypothalamus	3.646	2.9502	4.906	−1.5579
Commissural stria terminalis	3.1539	2.409	4.3944	−1.0779
Dorsal thalamus lateral nucleus group	3.4855	2.5493	5.0288	−1.5579
Dorsal thalamus	3.4855	2.5493	5.0288	−1.5579
Internal capsule	3.894	3.0805	4.7464	−1.3179
Nucleus around the septal area	3.1603	2.546	4.2774	−1.3179
Striatum	3.6427	3.0772	4.433	−1.0779

The significance was reflected by the Max_T. “*X,Y,Z*” represented the peak coordinates of the brain region's maximum effect point in Paxinos and Watson space, where the *x*-axis was midline with positive values on the right, the *y*-axis was positive ventrally and negative dorsally, and the *z*-axis was positive relative to the olfactory bulbs and negative towards the cerebellum.

**Table 4:** Changes in cerebral glucose metabolism between the Sham-EA group and the SHR group.

Anatomical	Max-T	Peak coordinates (mm)		
		*X*	*Y*	*Z*
Increased cerebral glucose metabolism				
Agranular insular cortex	4.5574	3.4353	6.5157	2.0421
Striatum	5.1039	3.0376	6.8059	1.8021
Piriform cortex	5.2391	2.8974	6.9016	2.2821
Orbital cortex	4.3093	2.0592	4.1385	4.9221
Caudate putamen	5.21	3.0376	6.9519	1.8021
Basal ganglia	5.21	3.0376	6.9519	1.8021
Lateral orbital cortex	4.4544	2.8908	6.5669	2.7621
Sensory cortex	4.4742	3.6019	2.0636	−0.3579
Lateral Prefrontal cortex	4.5574	3.4353	6.5157	2.0421
Prefrontal cortex	4.5574	3.4353	6.5157	2.0421
Decreased cerebral glucose metabolism				
Thalamus	7.2411	−1.8461	8.0611	−12.3579
Medulla oblongata	7.3504	−1.983	7.886	−12.1179
Pontine tegmentum of pons	5.1456	−2.3906	7.6742	−11.6379

The significance was reflected by the Max_T. “*X,Y,Z*” represented the peak coordinates of the brain region's maximum effect point in Paxinos and Watson space, where the *x*-axis was midline with positive values on the right, the *y*-axis was positive ventrally and negative dorsally, and the *z*-axis was positive relative to the olfactory bulbs and negative towards the cerebellum.

**Table 5:** Changes in cerebral glucose metabolism between the EA group and the Sham-EA group.

Anatomical	Max-T	Peak coordinates (mm)		
		*X*	*Y*	*Z*
Increased cerebral glucose metabolism				
Entorhinal cortex	4.1442	−4.8357	3.3369	−8.7579
Subiculum	4.2594	−4.575	2.8717	−8.2779
Corpus callosum	3.6719	−4.5816	2.8291	−7.7979
Cerebellum posterior lobe	3.7078	−4.6922	3.9926	−9.4779
Hippocampus	3.6775	−4.1708	3.3542	−8.5179
Olfactory cortex	4.3953	−4.5717	3.4771	−8.5179
Visual cortex	3.9127	−4.7087	2.4259	−8.2779
Decreased cerebral glucose metabolism				
Basal ganglia	3.8497	1.9949	5.4542	−0.1179
Hypothalamus	4.3019	1.6006	5.6197	−0.5979
Commissural stria terminalis	4.2555	1.7342	5.6274	−0.5979
Internal capsule	4.1441	1.8645	5.4678	−0.3579
Motor cortex	3.878	2.2325	0.60518	2.0421
Nucleus around the septal area	4.3019	1.6006	5.6197	−0.5979
Sensory cortex	3.8779	3.625	0.46043	−2.0379
Striatum	4.0306	1.8612	5.4465	−0.1179

The significance was reflected by the Max_T. “*X,Y,Z*” represented the peak coordinates of the brain region's maximum effect point in Paxinos and Watson space, where the *x*-axis was midline with positive values on the right, the *y*-axis was positive ventrally and negative dorsally, and the *z*-axis was positive relative to the olfactory bulbs and negative towards the cerebellum.

### 3.3 The regulation of EA on DEGs of SHRs

**Figure 3:**
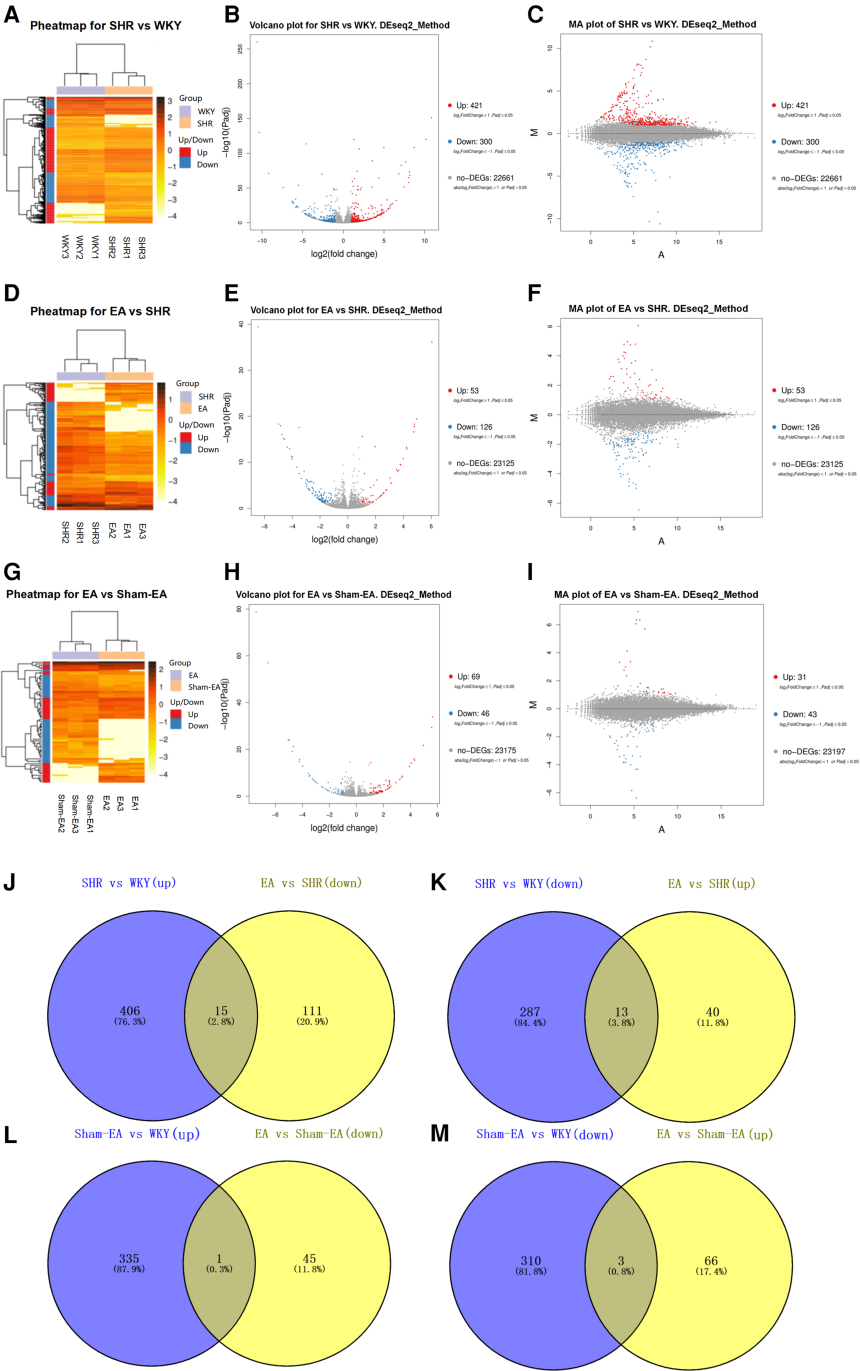
Significantly different mRNA expression in the hypothalamus of WKY, SHR, EA and sham-EA, *n* = 10. Hierarchical clustering (**A,D,G**), volcano plot (**B,E,H**) and MA plot (**C,F,I**) show that the differentially expressed genes (DEGs) between the 4 groups with red or blue colors suggesting upregulated or downregulated expression; (**J–M**) Venn diagrams showing overlapping DEGs between groups.

To investigate the regulation of EA on DEGs of SHRs, DEGs in the hypothalamus were analyzed. With the DESeq2 algorithm based on the negative binomial distribution, DEGs were detected by applying fold changes ≥2.00 and adjusted *p*-values (*p* ≤ 0.05). The statistics of the number of DEGs were obtained by heat map (Figures 3A,D,G), volcano plot (Figures 3B,E,H) and MA plot (Figures 3C,F,I) (The detailed DEGs can be found in Supplementary Tables S1–S4.). Compared to WKY rats, there were 721 DEGs in SHRs, including 421 up-regulated genes and 300 down-regulated genes. A total of 179 DEGs associated with SHR were found in EA rats, including 53 upregulated genes and 126 downregulated genes. Further, Venn analysis was used to study the genes that may lower BP due to acupuncture. We found that EA treatment repressed 15 DEGs out of 421 genes upregulated in SHR compared with WKY rats. Correspondingly, EA treatment was able to counteract 13 DEGs out of 300 downregulated genes in SHR (Figures 3J,K). Furthermore, compared to the Sham-EA group, EA was involved in 3 and 1 DEGs potentially upregulated or downregulated, respectively (Figures 3l,M).

Analyzing the DEGs regulated by EA or Sham-EA, we found that most of the DEGs were novel and unknown genes (see Supplementary Tables S5–S8), but only 11 DEGs (5 down-regulated, 6 up-regulated) were known genes regulated by EA (Tables 6, 7), and there was 1 DEG down-regulated by Sham-EA. Additionally, through further comparison, we found that the DEGs regulated by EA, compared to Sham-EA, were all unknown novel genes (see Supplementary Tables S9, S10).

**Table 6:** Known DEGs downregulated by EA treatment.

SHR vs. WKY (up) and EA vs. SHR (down)					
DEGs	Length	log2FoldChange	Padj	Up/down-regulation	*p* value
Xpot	3,510	−3.409860772	1.92 × 10^−09^	Down	1.81 × 10^−12^
Th	1,770	−1.678048015	0.033037048	Down	0.00061668
Trh	1,409	3.042834368	2.44 × 10^−07^	Down	4.40 × 10^−10^
Gpr88	4,164	1.567639036	0.031223836	Down	0.000569436
Nr4a1	2,503	1.213098741	2.83 × 10^−08^	Down	4.61 × 10^−11^

**Table 7:** Known DEGs upregulated by EA treatment.

SHR vs. WKY (down) and EA vs. SHR (up)					
DEGs	Length	log2FoldChange	Padj	Up/down-regulation	*p* value
Sirt1	944	−3.512874908	2.84 × 10^−18^	Up	9.76 × 10^−22^
Cck	702	1.335850166	0.013553003	Up	0.00017854
Atp9b	718	−1.931467332	5.76 × 10^−05^	Up	2.03 × 10^−07^
Phox2a	1,608	−1.840293434	0.004877082	Up	4.86 × 10^−05^
Htr5b	2,223	2.037956747	0.014162987	Up	0.00018901
LOC689064	637	1.443391964	0.009077454	Up	0.00010634

### 3.4 GO and KEGG analysis

**Figure 4:**
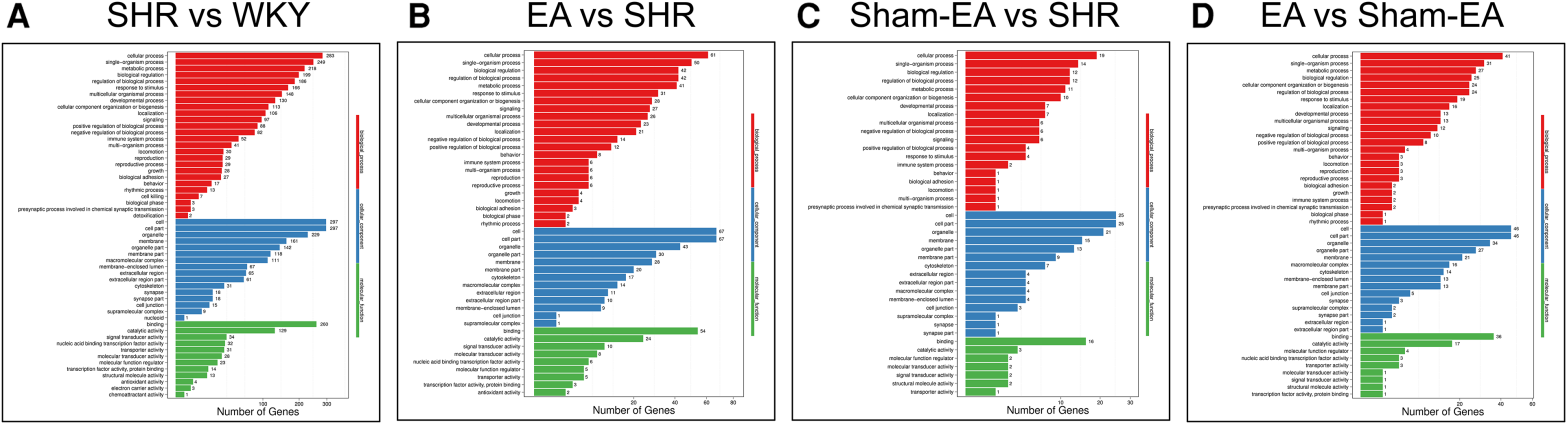
The most enriched gene ontology (GO) terms for the DEGs between WKY, SHR, EA and Sham-EA. **(A)** SHR vs. WKY, *n* = 10; **(B)** EA vs. SHR, *n* = 10; **(C)** Sham-EA vs. SHR, *n* = 10; **(D)** EA vs. Sham-EA, *n* = 10.

To investigate the function of the DEGs, Gene Ontology (GO) classification and functional enrichment were performed. GO covers three domains: biological process, cellular component, and molecular function. Functional enrichment was performed and the GO classification results between the WKY, SHR, EA and Sham-EA groups are shown in Figures 4A–D. Regarding biological processes, the categories “cellular process”, “single-organism process” and “metabolic process” showed a high degree of enrichment. The DEGs were involved in the “cellular process”, “single-organism process” and “biological regulation” categories according to their cellular component classification. In terms of the molecular functions, “molecular transducer activity”, “binding”, and “nucleic acid-binding transcription factor activity” showed a high degree of enrichment.

**Figure 5:**
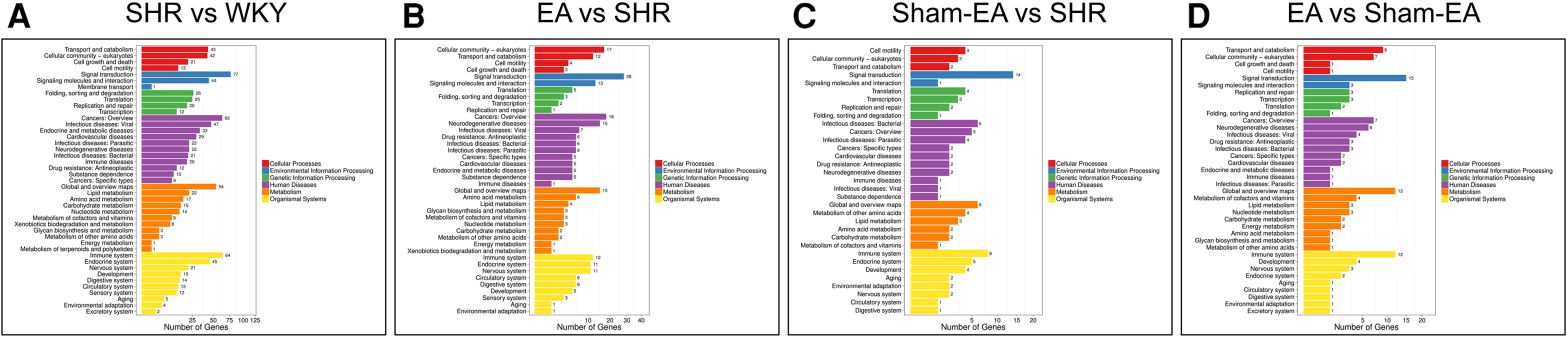
The DEGs number of the most enriched KEGG pathway functional results between WKY, SHR, EA and sham-EA. **(A)** SHR vs. WKY, *n* = 10; **(B)** EA vs. SHR, *n* = 10; **(C)** Sham-EA vs. SHR, *n* = 10; **(D)** EA vs. Sham-EA, *n* = 10.

To further investigate possible pathways directly affected by EA treatment in SHR, DEGs were classified by performing KEGG pathway classification and functional enrichment. Terms with a false discovery rate (FDR) no greater than 0.01 were defined as significantly enriched. As shown in Figure 5, DEGs were found to be enriched in several signaling pathways, including “neurodegenerative diseases”, “cardiovascular diseases”, “endocrine and metabolic diseases”, etc.

### 3.5 Validation of differentially expressed genes using real-time PCR

**Figure 6:**
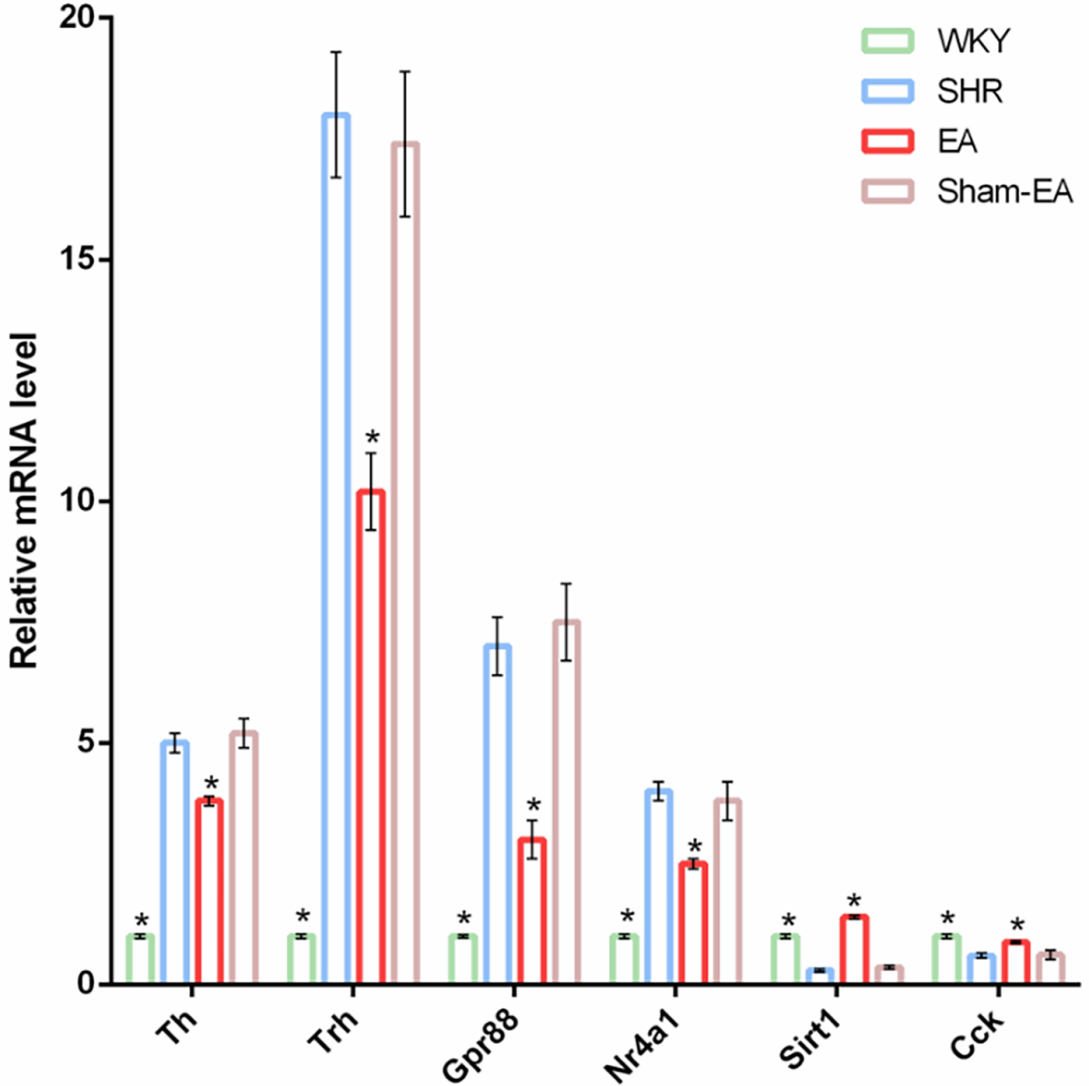
Verify DEGs up-regulated or down-regulated by EA using qRT-PCT. The loading control gene β-actin was used for normalization. Data were expressed as the mean ± SD and analyzed using ANOVA. *p < 0.05 vs. the SHR group, *n* = 3.

Before verifying potential known DEGs, only Nr4a1, Sirt1, Trh, GPR88, CCK, and Th were found to be related to the regulation of BP after reviewing the literature. To verify the reliability of the RNA-Seq data, real-time PCR was used to detect the expression of the six DEGs mentioned above. As shown in Figure 6, the trend of detected DEGs expression data was the same as that observed by RNA-Seq. Four genes Th, Trh, Gpr88 and Nr4a1 were upregulated in the SHR group but downregulated after EA treatment. On the other hand, 2 genes, Sirt1 and Cck, were downregulated in the SHR group but upregulated after EA treatment. However, Sham-EA did not significantly regulate these DEGs.

## 4 Discussion

Professor Deng Tietao has suggested that hyperactivity of liver yang is the main syndrome in the early stage of hypertension. LR3 is the Shu-stream point (Shu Xue) and Yuan-source point (Yuan Xue) of the liver meridian (30). Several clinical studies have shown that LR3 is an important acupuncture point for lowering BP (31–34).

Therapeutic efficacy distinguishes specific meridian points from sham acupoints. In this study, the depression between the third and fourth metatarsals on the dorsum of the foot was selected as the sham acupoint. This site is close to LR3, does not belong to any meridians, and has no antihypertensive effect.

Our study results indicate that the antihypertensive effect of EA at LR3 was stable and effective within the first 7 days of the treatment period. This was consistent with our previous research findings, demonstrating that both EA and manual acupuncture have antihypertensive effects and align with acupoint-specific characteristics (16, 35). Building upon this, our study further employed PET-CT scanning to identify the target brain regions affected by EA in the treatment of hypertension. The results revealed that, compared to the WKY group, glucose metabolism was upregulated in the dorsal thalamus, dorsal thalamus lateral nucleus group, and hypothalamus of the SHR group, and EA could reverse the glucose metabolism in these brain regions. However, Sham-EA could only modulate the glucose metabolism in the hypothalamus, not the dorsal thalamus. This suggested that the hypothalamus may be the specific target brain region of EA at LR3.

The hypothalamus is a central structure of the brain that provides adaptive, integrative, autonomous, and neuroendocrine responses to fluctuations in physiological conditions from external or internal environments (36). Dysfunction of the hypothalamus can lead to severe metabolic and functional disorders, including persistent elevation of BP. Therefore, the hypothalamus is considered a brain region closely associated with BP regulation (37, 38). The hypothalamus, in conjunction with the brainstem, regulates sympathetic nerve activity, often linked to the occurrence of primary hypertension (36). Studies have found that offspring of mice fed a low-protein diet during pregnancy exhibit salt-sensitive hypertension, associated with abnormal DNA methylation of the gene encoding angiotensin II receptor type 1A (AT1AR) in the hypothalamus (39). Moreover, activation of different cell types in the hypothalamus under metabolic stress conditions can lead to chronic, low-grade inflammation, disrupting energy balance and resulting in various diseases, including hypertension, diabetes, and obesity (40).

Our study results suggested that the hypothalamus was a crucial target brain region regulated by EA at LR3 in BP reduction. However, the specific molecular mechanisms were not yet clear. Therefore, we conducted mRNA expression profiling analysis on the hypothalamus of SHRs. The results showed that, compared to WKY rats, among the 421 upregulated DEGs in the hypothalamus of SHR rats, 15 could be reversed by EA; correspondingly, among the 300 downregulated DEGs, 13 could be reversed by EA. After excluding unknown and new genes, we conducted a literature search on the 11 known functional DEGs, determining that 6 of them—*Th*, *Trh*, *Gpr88*, *Nr4a1*, *Sirt1*, and *Cck*—were related to BP regulation. These genes were validated using qPCR. Therefore, combining our study results with literature reports, we speculated that the central molecular mechanisms of EA at LR3 in BP reduction were closely associated with the regulation of these target genes.

Tyrosine hydroxylase (TH) is widely expressed in the brain, including in the hypothalamus, substantia nigra, and amygdala, which play a role in regulating cardiovascular functions (41). TH is a key component in the development of high BP in SHRs. Researchers have detected higher levels of TH mRNA in the adrenal glands and medulla of SHRs compared to WKY (42). It is known that TH catalyzes the conversion of l-tyrosine to l-3,4-dihydroxyphenylalanine (L-DOPA), which is the initial and rate-limiting step in the biosynthesis of catecholamines, including dopamine, norepinephrine, and epinephrine (43, 44). Studies have found that high levels of TH increase the production of catecholamines, especially promoting the release of norepinephrine at the terminals of sympathetic nerve fibers, thereby enhancing sympathetic nervous activity and BP (45). It is reported that TH expression is regulated by complex short-term and long-term mechanisms. In a high-salt diet-induced hypertension rat model, TH and norepinephrine expression increase in the PVN of the hypothalamus, and antioxidant infusion can reverse these changes (46). Furthermore, researchers have found that resveratrol, by reducing TH expression and reactive oxygen species (ROS) levels, restores the balance of excitatory and inhibitory neurotransmitters, thereby alleviating hypertension (47). This study discovered that EA at LR3 can reduce the abnormally elevated TH in the hypothalamus of SHRs. Therefore, it is hypothesized that EA inhibits TH and reduces the production of catecholamines, especially norepinephrine, in the hypothalamus, thus suppressing sympathetic nervous activity and lowering BP.

Silencing type information regulation 2 homolog-1 (SIRT1) is a deacetylase expressed in various locations, including the PVN (48) and vascular endothelial cells (49). Researchers have found that the overexpression of SIRT1 can protect mice from vascular remodeling and hypertension induced by angiotensin II (Ang II) (50). It is noteworthy that SIRT1 protects the homeostasis of endothelial cells and blood vessels by influencing the neural inflammatory response and oxidative stress, thereby potentially exerting its BP-lowering effects (51–53). Highly activated NF-*κ*B is a major regulatory factor in sympathetic nervous activity, and inhibiting NF-*κ*B in the PVN can reduce inflammation and oxidative stress, thereby affecting sympathetic nerve activity and BP (54). It has been reported that SIRT1 can regulate nuclear factor *κ*B (NF-*κ*B) to interfere with the development of neurons, maintain normal neural function, and protect neurons (55, 56). Upregulating the expression of SIRT1 in the PVN of SHRs reduces NF-*κ*B p65 activity, inhibits inflammasome formation, thereby weakening sympathetic nervous activity, and alleviating hypertension. Conversely, the knockout or loss of SIRT1 weakens its effects, leading to excessive acetylation of NF-*κ*B p65, increased expression of NOX4 and ROS, and enhanced oxidative stress (47). The results of this study suggest that EA can upregulated SIRT1 expression in SHR. We hypothesized that EA may alleviate neuroinflammation and oxidative stress, reduce sympathetic outflow, and alleviate hypertension by inhibiting SIRT1 expression.

NR4A1 is a member of the nuclear hormone receptor superfamily, a class of ligand-dependent transcription factors that are distributed intracellularly and regulate gene transcription and expression by binding to specific ligands. However, due to the unidentified ligand, it is also known as an orphan nuclear receptor (57, 58). NR4A1 is involved in various physiological processes, including inflammation, cell differentiation, apoptosis, proliferation, and metabolism (59). Research indicates that NR4A1 plays a role in activating neuroinflammation in the central nervous system. Wu et al. found that in an animal model of ischemic brain injury, the expression of NR4A1 is elevated, leading to M1 polarization and neutrophil aggregation by activating p65 (60, 61). Additionally, in a mouse traumatic brain injury model, the deletion of CX3C chemokine receptor 1 induces the expression of clusterin 36 and 15-lipoxygenase, followed by an increase in NR4A1 expression, thereby exacerbating brain injury (62). Overall, increased NR4A1 expression promotes neuroinflammation. It is worth noting that neuroinflammation is considered a key factor in enhancing hypertension and sympathetic nervous activity (63). Researchers have found that intraventricular injection of the pro-inflammatory cytokine IL-1β in the hypothalamus leads to increased BP (64), and neural inflammation in the hypothalamus has been shown to be positively correlated with neurogenic hypertension (65). This study found that NR4A1 is abnormally elevated in the hypothalamus of SHR, and EA treatment reduced NR4A1 expression. Therefore, we speculated that NR4A1 may be a potential target for acupuncture treatment of hypertension, and acupuncture may achieve this by inhibiting central neuroinflammation.

As is well known, the molecular mechanisms underlying the hypotensive effects of acupuncture exhibit characteristics of multiple targets and pathways. In addition to the aforementioned mechanisms, this study reveals that acupuncture also regulates the expression of GPR88, TRH, and CCK in the hypothalamus. GPR88 is an orphan G protein-coupled receptor primarily expressed in the brain, heart, and kidneys (66, 67). Current research indicates that the loss of central GPR88 gene expression is associated with dopamine system imbalance (68). It has been reported that GPR88 may influence the cardiovascular system and regulate BP by modulating dopamine (67). However, the specific pathways through which acupuncture upregulates GPR88 expression to achieve hypotensive effects remain to be further elucidated. Furthermore, thyrotropin-releasing hormone (TRH) has a central pressor effect. Researchers have found that the pressor effect of TRH is partly mediated by central *α*1 receptors, and its peripheral mechanism primarily involves exciting the sympathetic nervous system, causing peripheral blood vessel constriction. It also activates β receptors in juxtaglomerular cells, promoting renin release, leading to an increase in BP (69). Cholecystokinin (CCK) is a typical brain-gut peptide hormone, primarily composed of amino acid peptides, and mainly exists in the central nervous system in the form of CCK8. CCK, along with gastrin and somatostatin, is a stress-sensitive hormone (70). Research indicates that CCK can convey signals from the vagus nerve to the hypothalamus through the solitary tract nucleus in the brainstem (71), which may be closely related to the body's stress response. Cholecystokinin (CCK) is considered one of the environmental factors regulating hypertension. Changes in CCK during stress provide a new avenue for analyzing the pathogenesis of hypertension.

Finally, it is worth noting that our study used the Sham-EA group as a sham-acupoint control to demonstrate the acupoint-specific effects of acupuncture on hypertension. In this study, compared to the EA group, the Sham-EA group did not exhibit an effective antihypertensive effect. And, further brain functional imaging technology PET-CT scans showed that the brain regions regulated by Sham-EA were not related or directly related to lowering BP. Additionally, the results of RT-PCR suggested that Sham-EA intervention had no significant upregulation or downregulation effect on the abnormally expressed DEGs. Taken together, these data indicate that the sham-acupoint, as a non-meridian acupoint, has no therapeutic effect, highlighting the specific therapeutic efficacy of the LR3 acupoint.

## 5 Conclusion

In summary, this study indicated that EA at the LR3 can effectively reduce BP in SHRs. This effect may be achieved through improving neuroinflammation, inhibiting oxidative stress responses, and attenuating sympathetic nervous system overactivity in SHRs. Our research results provided evidence for the efficacy of acupuncture in treating hypertension and established a foundation for further exploring the central mechanisms of acupuncture-induced BP reduction.

## Data Availability

The original contributions presented in the study are publicly available. This data can be found here: http://www.ncbi.nlm.nih.gov/bioproject/1147573.
